# Simple change in logistic procedure improves response rate to QOL assessment: a report from the Japan Children’s Cancer Group

**DOI:** 10.1186/s41687-020-00214-9

**Published:** 2020-06-17

**Authors:** Iori Sato, Takafumi Soejima, Yasushi Ishida, Miho Maeda, Katsuyoshi Koh, Kiyoko Kamibeppu

**Affiliations:** 1 Center for Quality of Life Research, The University of Tokyo, Bunkyo-ku, Tokyo, Japan; 2 Department of Family Nursing, Division of Health Science and Nursing, Graduate School of Medicine, The University of Tokyo, Bunkyo-ku, Tokyo, Japan; 3 Committee on Long-term Follow-up, Japan Children’s Cancer Group, Nagoya, Aichi Japan; 4 Pediatric Medical Center, Ehime Prefectural Central Hospital, Matsuyama, Ehime Japan; 5 Department of Pediatrics, Nippon Medical School, Bunkyo-ku, Tokyo, Japan; 6 Committee on Acute Lymphoblastic Leukemia, Japan Children’s Cancer Group, Nagoya, Aichi Japan; 7 Department of Hematology/Oncology, Saitama Children’s Medical Center, Saitama, Japan

## Abstract

**Background:**

Reducing non-completion of quality-of-life assessment in clinical trials is an important challenge in obtaining accurate data and unbiased interpretation of patients’ quality-of-life for each regimen. We evaluated the effect of changing our questionnaire distribution procedure in a multicenter phase II/III trial on the response rate to a quality-of-life questionnaire.

**Methods:**

In the trial, we distributed 1767 questionnaires and 1045 were returned. We adopted a regression discontinuing design and estimated the change in response rate between pre-intervention (quality-of-life questionnaires were sent to each center soon after patient registration) and post-intervention (a set of tailored questionnaires was sent just before the first quality-of-life assessment).

**Results:**

The post-intervention response rate was higher (odds ratio = 1.62) than the pre-intervention response rate.

**Conclusions:**

A simple logistic intervention reduced the non-completion of QOL assessment in this case, suggesting that a simple change can contribute to improving clinical trial accomplishment.

## Background

Patient-reported outcome (PRO) is an important endpoint in clinical trials [1]. Obtaining PRO data requires patient cooperation, including in trials involving children. In multicenter trials, physicians or research coordinators at each center distribute PRO questionnaires at specified time-points to patients. Many factors contribute to whether patients’ PRO data are ultimately obtained [2]. To date, however, few reports have examined strategies for reducing non-completion of PRO assessment.

When a patient is registered in a trial with quality of life (QOL) assessment in the Japanese Pediatric Leukemia/Lymphoma Study Group (JPLSG), later changed to the Japan Children’s Cancer Group, the QOL office generates a set of age-appropriate QOL questionnaires and return envelopes, and sends them to a physician in the corresponding center soon after patient registration. The physician or research coordinator subsequently distributes the questionnaire and envelope to the patient at the required time-point for QOL assessment. Patients respond to and return the questionnaire directly to the QOL office.

Background factors in this process are as follows. Patients return the questionnaire directly and not through their clinical provider for ethical considerations - specifically, we were concerned that they would find it difficult to respond candidly to the assessment questions (eg. *It is hard for me to tell the doctors and nurses how I feel*) if the doctors and nurses were involved in collection. Further, the QOL office is unable to serve the questionnaire directly to patients for reasons of personal information protection, and centers cannot share participant e-mail or postal addresses, even for clinical trials. Finally, our budget did not permit the placement of non-clinical staff for collection at each center.

Here, we aimed to improve response rates to QOL assessment by focusing on logistic factors [2]; in particular, the distribution timing of questionnaires.

## Methods

We conducted an intervention for JPLSG ALL-B12 (jRCTs041180101, UMIN000009339), a multicenter phase II/III study in children aged 1–19 years with newly diagnosed B-cell precursor acute lymphoblastic leukemia. Treatment regimens were all based on a BFM backbone. Primary endpoint was 5-year event-free survival and secondary endpoints included patient- and parent-reported QOL, measured using the Pediatric Quality of Life Inventory Generic Core Scales [3, 4], Cancer Module [5, 6] and Multidimensional Fatigue Scales [6, 7]. QOL was assessed at the end of (1) induction therapy (about 6 weeks after treatment initiation), (2) a consolidation therapy (about 21 weeks after treatment initiation), (3) all consolidation therapy (about 1 year after treatment initiation), (4) maintenance therapy (about 2 years after treatment initiation) and (5) 1 year after the end of all therapy (about 3 years after treatment initiation). This study was conducted utilizing only the first assessment. Exact time from treatment initiation varies because therapy was extendable based on patient condition. Registration period was November 2012 to December 2017.

In July 2016, we changed the distribution procedure for QOL questionnaires. Conventionally, a physician receives the questionnaire soon after treatment initiation (1–2 weeks in practice) and distributes it around the sixth week. We speculated that this procedure left physicians vulnerable to misplacing the questionnaire or forgetting to conduct the QOL assessment. We therefore decided to send the questionnaire to physicians in the fifth week. We announced this change at a meeting with participating centers in June 2016 and initiated the change the following month (July 2016). We hypothesized that the QOL questionnaires would more likely reach patients/parents using the new distribution procedure, in turn increasing the response rate. However, procedure changes can cause confusion at participating centers. We therefore evaluated the effectiveness of this change based on response rate to the first QOL assessment.

ALL-B12 was conducted with the approval of the National Hospital Organization Review Board for Clinical Trials (Nagoya) (C2018–001). Children and/or their parents gave written informed consent to ALL-B12. Because they returned the questionnaire directly to the QOL office, their attending physicians did not know whether the patient responded or not; therefore, the need for the physician to compel a response was obviated.

## Results

Among ALL-B12 participants, QOL assessment was only conducted in children aged 2–18 years at each assessment time-point. Through June 2016 (43 months), before the procedure change, we sent 557 (earlier 22 months) and 676 (later 21 months) sets of the first QOL questionnaire to the 134 participating centers, and 381 and 367 questionnaires were returned (response rate = 68% and 54%), respectively. From July 2016 (19 months), after implementing the procedure change, we sent out 534 sets of the first QOL questionnaire, and 297 were returned (response rate = 56%). These results are detailed by month in Supplementary Figure 1.

**Fig. 1:**
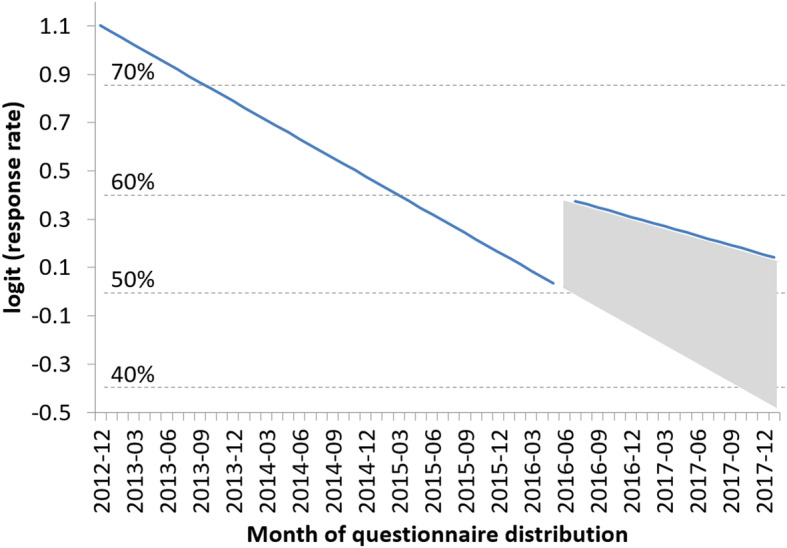
Trend of response of questionnaire by month

Generalized linear mixed models (Table 1) showed that the response rate decreased with time (1-year OR = 0.74); however, the response rate in July 2016 was significantly higher than that in the preceding month (OR = 1.62). Regression discontinuity design [8] showed a similar difference in response rates (OR = 1.47). Further, the negative slope of the response rate was shallower after implementing the intervention (Model 4 in Table 1, Fig. 1).

**Table 1:** Estimated effect on response to QOL questionnaire (*N* = 1767)

	Model 1^a^		Model 2^b^		Model 3^c^		Model 4^d^		Model 5^e^	
	AOR	95% CI	AOR	95% CI	AOR	95% CI	AOR	95% CI	AOR	95% CI
After the change in procedure	0.79	0.63 to 1.00	1.64	1.13 to 2.38	1.62	1.11 to 2.35	1.47	0.93 to 2.32	1.47	0.93 to 2.32
Timing of distribution (per 1 year)	–	–	0.74	0.65 to 0.84	0.74	0.65 to 0.84	–	–	–	–
Timing of distribution (per 1 year) before the change in procedure	–	–	–	–	–	–	0.73	0.64 to 0.84	0.73	0.64 to 0.84
Timing of distribution (per 1 year) after the change in procedure	–	–	–	–	–	–	0.86	0.56 to 1.31	0.86	0.56 to 1.32
Child’s age (per 1 years old)	–	–	–	–	0.96	0.94 to 0.99	0.96	0.94 to 0.99	0.96	0.94 to 0.99
Hospital volume (per 10 cases registered in ALL-B12)	–	–	–	–	0.97	0.82 to 1.16	–	–	0.98	0.82 to 1.17

*AOR* adjusted odds ratio, *CI* confidence interval, *QOL* quality of life

^a–e^Dependent variable: QOL questionnaires that received a response (1) or no response (0)

^a–e^Random effect: each center of JPLSG (number of centers = 134)

^a–c^Generalized linear mixed model

^d–e^Regression discontinuity design

As post-hoc analysis, we estimated the increase in questionnaires returned due to implementing the intervention (shaded trapezoidal area in Fig. 1). Accordingly, if the procedure for sending QOL questionnaires had not been changed and the decrease in response rate had continued, an estimated 233 of the 534 questionnaires sent after July 2016 would have been returned (counterfactual response rate = 44%). Bootstrap estimation predicts that an extra 64 questionnaires (95% confidence interval = 16–111) were returned due to the intervention.

## Discussion

This study reports the effectiveness of a simple logistic intervention in reducing the non-completion of QOL assessment. The intervention consisted of simply changing the timing of distribution of questionnaires to participating centers from the first/second to the fifth week after treatment initiation. This simple intervention clearly improved the questionnaire response rate. We therefore plan to adopt this distribution procedure in future JPLSG protocols.

The new procedure is also expected to reduce the burden on physicians, who no longer need to keep the QOL questionnaire for a period of weeks but can instead administer it to patients almost immediately after receipt. Inconvenience and lack of manpower are barriers to introducing PRO assessment [9]. Our results clearly indicate that physician misplacement of the questionnaires or forgetting to conduct the QOL assessment is a major factor in non-completion. Determining the feasibility of different procedures for each center is important, as is consideration for manpower at the QOL office.

Changing the procedure in the middle of the clinical trial did not seem to cause confusion in the centers. In contrast, the QOL office received only a few reminders from some of the centers stating, “We have not received the QOL questionnaire set for a patient. Have you already sent it?” In response, the QOL office immediately sent the questionnaire set to the inquiring center. No obvious adverse effects arose from the procedure change.

It is possible that not all of the estimated effect of the intervention was directly due to the change in distribution procedure. For example, the change may have increased some physicians’ interest in QOL assessment. However, we do not think that awareness of QOL (i.e. understanding of the importance of QOL assessment) is easily changed. We announced this change in procedure only once, and informed staff only about what would be changed (timing of distribution), not why the change was being made (i.e. to improve the response rate). It is therefore unlikely that this announcement and change in procedure increased interest in QOL assessment at the participating centers. Instead, we propose that the estimated effect was due to the change in distribution procedure.

This intervention did not strongly appeal or intrusively compel the centers to administer the QOL questionnaire to patients. Thus, it functioned as a ‘nudge’ intervention [10]. The change in procedure likely induced an unconscious change in physicians’ behavior. Our findings indicate that even a casual change in clinical trial design can lead to a significant improvement.

In the current setting, utilization of electronic data collection systems is feasible. At the time we introduced QOL assessment into clinical trials around 2010, however, we were unable to determine whether QOL assessment would be routinely included in future clinical trials at all JPLSG centers and, given the high initial cost of electronic systems, selected a paper-based system instead. With our current implementation of QOL assessment in several trials, however, the total cost of an electronic system in supplies and manpower would be lower than the present paper-based system, and implementation of an electronic data collection system for JPLSG is now underway. With these systems, the initial distribution of system information to patients should be done by a physician or research coordinator at each center. Therefore, the implications of this study concerning distribution process and timing are still relevant.

Some limitations of this study warrant mention. First, we only reported the effect of the intervention on the response rate to the first QOL questionnaire. A previous paper reported that while it was possible to improve the response rate, sustaining the improvement is difficult [11]. Second, the reason for the decrease in response rate with time is unclear. Reported factors for non-completion of QOL assessment [2] do not explain the decrease. Third, we did not employ a randomized design, and the possibility of confounding by unknown confounding factors cannot be eliminated.

These limitations notwithstanding, we found that a seemingly minor change in the distribution timing of questionnaires resulted in a clinically significant improvement in first QOL assessment in a clinical trial of pediatric cancer.

## Conclusions

This study reports the effectiveness of a simple logistic intervention in reducing the non-completion of QOL assessment. Such a simple intervention clearly improved the questionnaire response rate in this case. A simple change can contribute to improving clinical trial accomplishment.

## Supplementary information

**Additional file 1.**

## Data Availability

The datasets generated and/or analyzed during the current study are not publicly available because the ALL-B12 trial is no longer recruiting but continuing follow-up. QOL survey at the end of induction therapy was completed and the data of this study is confirmed, meanwhile, the main body of ALL-B12 is not completed. However, these are available from the corresponding author on reasonable request.

## Abbreviations

AOR: Adjusted odds ratio
CI: Confidence interval
JPLSG: The Japanese Pediatric Leukemia/Lymphoma Study Group
OR: Odds ratio
PRO: Patient-reported outcome
QOL: Quality of life
