# Supplementary figures and images for: Simple change in logistic procedure improves response rate to QOL assessment: a report from the Japan Children’s Cancer Group
Source: J Patient Rep Outcomes. 2020 Jun 17;4:48. doi: 10.1186/s41687-020-00214-9 (PMC7300165; doi:10.1186/s41687-020-00214-9)

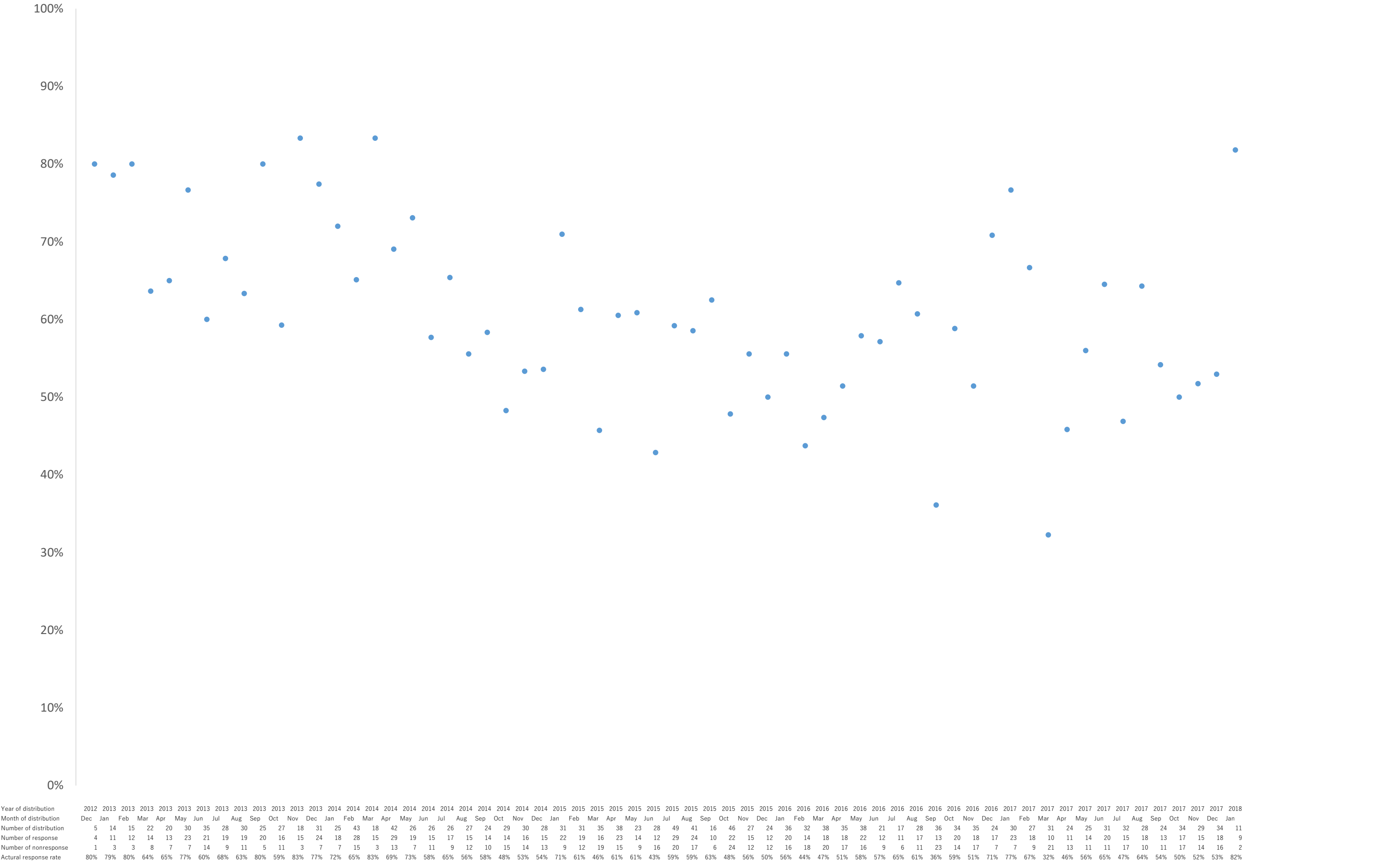

Supplement: Supplementary file 1 — Additional file 1. [file 41687_2020_214_MOESM1_ESM.pdf]
